# Valorization of Polysaccharides Obtained from Dark Tea: Preparation, Physicochemical, Antioxidant, and Hypoglycemic Properties

**DOI:** 10.3390/foods10102276

**Published:** 2021-09-26

**Authors:** Jiangxiong Zhu, Hui Zhou, Junyao Zhang, Fanglan Li, Kang Wei, Xinlin Wei, Yuanfeng Wang

**Affiliations:** 1 Development Center of Plant Germplasm Resources, College of Life Sciences, Shanghai Normal University, 100 Guilin Road, Xuhui District, Shanghai 200234, China; zjx261023@163.com (J.Z.); zhouhui1094@163.com (H.Z.); 13183033753@163.com (J.Z.); 18623706069@163.com (F.L.); 2 Department of Food Science & Technology, School of Agriculture and Biology, Shanghai Jiao Tong University, 800 Dongchuan Road, Minhang District, Shanghai 200240, China; wk5516828@sjtu.edu.cn

**Keywords:** dark tea polysaccharides, physicochemical composition, structural characteristics, antioxidant activities, hypoglycemic activities

## Abstract

The structure and hypoglycemic activity of tea polysaccharides has been extensively studied, while there are few reports on the characterization and hypoglycemic activity of dark tea polysaccharides. The crude dark tea polysaccharide (CDTPS) was optimally extracted from Fuzhuan dark tea. Six polysaccharide fractions (namely DTPS-1, DTPS-2, DTPS-3, DTPS-4, DTPS-5, and DTPS-6) were isolated from CDTPS, and their physicochemical, structural, and biological properties were compared and analyzed. The results revealed that the compositions, structural characteristics, and biological properties of the six DTPSs were different. Therein, DTPS-4 and DTPS-6 had looser morphology, faster solubility, and a more stable structure. Additionally, DTPS-4 had the optimum in vitro antioxidant capabilities, and DTPS-6 had the strongest in vitro hypoglycemic capabilities. In addition, a correlation analysis revealed that the molecular weight and uronic acid content were significantly related to their antioxidant and hypoglycemic activities. Our results indicated that DTPS-4 and DTPS-6 could be further developed into functional foods or additives, respectively.

## 1. Introduction

Tea is the second-largest consumed beverage in the world after water, with a long history. According to different manufacturing processes and characteristics, tea can be roughly divided into six categories, including green tea, black tea, dark tea, yellow tea, oolong tea, and white tea [1]. Dark tea is a unique post-fermented tea that is fermented by microorganisms, and the source of its tea leaves is relatively coarse and old [2]. Compared with green tea and black tea, dark tea has received less attention. As a kind of dark tea, Fuzhuan tea is made from coarse tea leaves and is a post-fermented brick-style tea produced by fermentation of the unique fungus *Eurotium cristatum*. It has been proven to exhibit preventive and therapeutic effects on many chronic metabolic diseases, especially those related to glucose and lipid metabolism [3,4,5]. However, there are still few studies on Fuzhuan tea. In East Asian countries, such as China and Japan, coarse tea has traditionally been used to treat diabetes as a popular folk prescription, which can be associated with tea polysaccharides (TPS) [6]. TPS, one of the representative functional components of tea, has been reported for enhancing immunity and resisting radiation and tumors, especially reducing the risk of diabetes [7,8,9,10]; its proportion in coarse tea is higher than that in other ordinary teas [11]. At present, TPS has received much less attention than tea catechins and polyphenols, because most studies have focused on the physicochemical properties and bioactivities of components with low molecular weight (Mw) in tea, such as catechin and caffeine [10]. Many in vivo and in vitro experimental evidence have shown that TPS can inhibit α-glucosidase and α-amylase activities, ameliorate insulin resistance, improve glucose tolerance, and reduce the blood glucose level [12,13,14,15]. In addition, there have also been many reports on the hypoglycemic activity of DTPS. The research by Deng et al. showed that Pu-erh DTPSs could significantly reduce the blood glucose of mice fed with starch through the inhibition of α-glucosidase activity [15]. Chen et al. found that Fuzhuan DTPSs alleviated the disorder of lipid metabolism of mice induced by a high-fat diet [3]. In addition to antidiabetic activity, it is reported that TPS also has good antioxidant effects. Many in vivo experiments have shown that tea polysaccharides could reduce blood glucose by enhancing antioxidant capacity [3,16]. Similarly, the antioxidant activity of DTPS has also been widely studied. Studies have indicated that Fuzhuan DTPSs had a good capability to scavenge a variety of free radicals [17]. Qingzhuan DTPSs have been reported to possess good free radical scavenging and absorption capacities and ferric reducing activity [18]. These all reveal that DTPS can play a role in decreasing blood glucose via a variety of mechanisms and is expected to be further developed as a potential candidate substance for preventing and alleviating diabetes.

Our previous study found that TPS from green tea exhibited α-glucosidase and α-amylase inhibitory activities, while the physicochemical properties and antidiabetic activities of TPS from different resources and extraction processes were quite different, and this could be explained, in part, by their diversity in chemical composition and structural characteristics [19]. Therefore, it is necessary to investigate the structural properties of DTPSs to clarify their blood glucose-lowering mechanism and apply them in the food industry.

In this study, a novel DTPS was prepared and isolated, and the physicochemical and structural features of isolated polysaccharide fractions were explored. Furthermore, the bioactivities, including in vitro antioxidant and hypoglycemic activities, were investigated, and the correlation between their physicochemical composition and in vitro antioxidant and hypoglycemic activities was analyzed. This paper further provides a reference for the potential utilization of DTPS in the prevention or management of diabetes and the development of functional food.

## 2. Materials and Methods

### 2.1. Materials and Reagents

The novel Fuzhuan dark tea (FZDT) (*Camellia L.*) growing up in the Hubei Province of China was harvested from Enshi selenium impression Agricultural Development Co., Ltd. (Hubei, China). The fresh green tea leaves picked in late spring were processed into raw dark green tea through a series of steps, such as fixation and pile fermentation. Then, the raw dark green tea (with a moisture content of about 20%) was steamed, pressed, fermented, and, finally, dried with hot air to obtain Fuzhuan dark tea. HepG2 cells were gifted from Shanghai Institutes for Biological Sciences, CAS (Shanghai, China). Diethylaminoethyl (DEAE) Sepharose Fast Flow gel was purchased from GE Co. (St. Louis, MO, USA). The glucose oxidase method kit was purchased from Applygen Technologies Inc. (Beijing, China). The dialysis bags (3500 and 12,000 Da) were obtained from Shanghai Solarbio Bioscience & Technology Co., Ltd. (Shanghai, China). Galacturonic acid was provided from Shanghai Sinopharm Chemical Reagent Co., Ltd. (Shanghai, China). Bovine serum albumin (BSA) and 2,2′-diazonium (3-ethylbenzothiazoline-6-sulfonic acid) diammonium salt (ABTS) were purchased from Aladdin (Shanghai, China). Alpha-glucosidase, alpha-amylase, p-nitrophenyl-D-PNPG glucoside (pNPG), and reduced glutathione were all provided from Shanghai Yuanye Bio-Technology Co., Ltd. (Shanghai, China). 1,1-diphenyl-2-picrylhydrazyl (DPPH) was purchased from Sigma Chemical Co., Ltd. (St. Louis, MO, USA), and 3-(4,5-dimethyl-2-thiazolyl)-2,5-diphenyl-2-H-tetrazolium bromide (MTT) was provided from Solarbio Bioscience & Technology Co., Ltd. (Beijing, China). The DMEM medium was obtained from HyClone Co., Ltd. (Los Angeles, CA, USA), and the fetal bovine serum (FBS) and 0.25% Trypsin-EDTA were purchased from Gibco Co., Ltd. (Carlsbad, CA, USA). All other chemicals and solvents were of analytical grade.

### 2.2. Single-Factor and Response Surface Methodologies

The dried FZDT leaves were pulverized and then pretreated with 85% ethanol twice at 25 °C for 8 h to remove some small molecules. After filtration, the residues were dried; extracted with distilled water twice (the water to solid ratio was 1:15) at a range of extraction temperatures (50, 60, 70, 80, 90, and 100 °C) for 2 h; and then centrifuged at 5000× *g* for 10 min. The supernatant was collected, concentrated to 1/3 of its original volume using a rotary evaporator, and precipitated by the 80% ethanol of equal volume and stored overnight at 4 °C. The resultant precipitates were collected by centrifugation, resuspended in distilled water, and treated with 15% trichloroacetic acid to remove proteins. Subsequently, the aqueous phase was dialyzed (cutoff Mw 3500 Da) for 48 h to remove small molecular compounds and inorganic salts. After lyophilization, the CDTPS was obtained, and its yield was measured. Similarly, the other same conditions were kept, except for the extraction times (0.5, 1, 1.5, 2, and 2.5 h), and the CDTPS yield was measured again. In a like manner, the other same conditions were kept except for the solid–liquid ratio (1:5, 1:10, 1:15, 1:20, and 1:25), and the CDTPS yield was measured again.

Based on single-factor experiments, response surface methodology was performed to analyze the effects of the extraction temperature, extraction time, and solid–liquid ratio on the CDTPS yield (Design-Expert 8.0.6 Trial, State-Ease, Inc., Minneapolis, MN, USA). According to the Central Composite Design (CCD), the three-factor and three-level response surface methods were used, and 17 experiments were carried out. These factors and levels were presented in Table S1.

### 2.3. Isolation of Polysaccharides

The CDTPS (200 mg) was fractionated by DEAE Sepharose Fast Flow gel on a column (2.5 × 60 cm). The sample was dissolved in 5 mL of 0.02-M PBS (pH = 6.0) and then centrifuged at 5000× *g* for 15 min, and the supernatant was passed through a 0.45-μm membrane; then, it was eluted with a PBS buffer solution containing different concentrations (0, 0.1, 0.2, 0.3, 0.4, and 0.5 M) of a NaCl solution at a flow rate of 2 mL/min. Eluted fractions corresponding to the peaks were collected under monitoring by the phenol-sulfuric acid method, dialyzed against distilled water, and lyophilized.

### 2.4. Preliminary Chemical Analysis

The neutral polysaccharide content was measured by the 3,5-dinitrosalicylic acid colorimetric method using the procedure of the national agricultural industry standard of China (NY/T2742-2015). The Kjeldahl method regarding the national standard method of China (GB/5009.5-2010) was performed to determine the soluble protein content. The uronic acid content was determined by the meta-hydroxyphenyl method [20]. The polyphenol contents were determined by the Folin–Ciocalteu method regarding the national standard method of China (GBT8313-2018). The standard curves of the corresponding standards are shown in Figure S2.

### 2.5. Physicochemical Composition and Structural Characterization

#### 2.5.1. Monosaccharide Composition and Molecular Weight Analysis

The monosaccharide composition of the sample was analyzed according to the report described by our lab [21]. Briefly, the polysaccharide sample (10 mg) was dissolved in 4 mL of 2-M trifluoroacetic acid (TFA) solution and hydrolyzed under nitrogen at 120 °C for 4 h. After concentration, methanol was added to the hydrolysate, and the rotary evaporation was repeated until the TFA was completely removed. Next, deionized water was added and diluted to 100 mL for ion chromatography (IC) measurement. A total of 25 μL of the treated sample solution was injected into a Dionex CarboPac PA20 anion exchange column (150 mm × 3 mm) with the temperature maintained at 30 °C and eluted with 2-mM NaOH at a flow rate of 0.45 mL/min. Monosaccharide standards, including fucose, rhamnose, arabinose, glucosamine, galactose, glucose, mannose, xylose, fructose, GalA, and GluA, were measured by the same procedure. The molecular weight of the polysaccharide samples was determined according to the report by our lab [21].

#### 2.5.2. Ultraviolet–Visible (UV–Vis) and Fourier-Transform Infrared (FTIR) Analysis

A scanning analysis of the sample (80 μg/mL) was performed using the UV–Vis spectrophotometer (Pussy General Instruments, Beijing, China) in the range of 190–500 nm.

The dried sample (2 mg) was crushed and mixed with KBr, pressed into a 1-mm-thick transparent and uniform pellet, and measured by an Avatar-360 FTIR spectrometer (Thermo Nicolet, MA, USA) and scanned in the range of 4000–500 cm^−1^.

#### 2.5.3. Scanning Electron Microscopy (SEM) Analysis

A total of 2 mg of sample was coated with a thin gold layer under reduced pressure using an E-1045 ion sputter coater (Ted Pella, CA, USA), the SEM analysis was performed by a HITACHSU8010 scanning electron microscope (JEOL, Tokyo, Japan), and the image magnification was 250×.

#### 2.5.4. Atomic Force Microscope (AFM) Analysis

A small amount of sample (50 μg/mL) was absorbed with a rubber-tipped dropper, placed on the mica plate, dried under the infrared lamp, and then detected on the MFP-3D AFM (Oxford Instruments, Shanghai, China).

#### 2.5.5. X-ray Diffraction (XRD) Analysis

The XRD of the sample was determined according to the method described by our lab [22]. The sample was measured by an X-ray diffractometer (X’Pert Pro MPD, PANalytica, Almelo, The Netherlands). The X-ray diffraction conditions were as follows: Cukα radiation, 35 kV of tube pressure, 100 mA of tube current, 10–70° angle, and 0.02° angle gradient.

#### 2.5.6. Thermogravimetric Analysis (TGA)

The thermal stability of the samples was analyzed by TGA using a Simultaneous thermal analyzer (NETZSCH STA 449F3, Selb, Free State of Bavaria, Germany). The sample (10 mg) was heated at a rate of 10 °C/min under the protection of nitrogen and observed in the range of 20–800 °C.

#### 2.5.7. Solubility and pH of DTPSs

The solubility and pH of the DTPSs were carried out according to the description reported by our lab [19]. The sample (50 mg) was added to 25 mL of distilled water and then placed in a water bath at different temperatures (20, 40, 60, 80, and 100 °C) with stirring at the same speed (500 rpm) until the dried sample was completely dissolved, and the dissolution time was recorded.

### 2.6. In Vitro Antioxidant Activities

#### 2.6.1. ABTS Radical Scavenging Assay

The ABTS radical scavenging activity was employed according to the previous method [23]. Briefly, a 7-mM ABTS and 2.45-mM K_2_S_2_O_8_ solution were mixed in equal volume and stood for 12–16 h under the dark condition at room temperature. Then, PBS (0.2 M, pH = 7.4) was diluted to make the absorbance reach 0.70 ± 0.02 at 734 nm to form an ABTS solution. After that, 4.0 mL of ABTS was mixed fully with 100 μL of the sample solution (25, 50, 100, 200, and 400 μg/mL) and was then measured at 734 nm. The PBS was used as the blank group instead of the sample, while the control group replaced the ABTS solution with PBS.

ABTS radical scavenging activity (%) = [A0 − (A1 − A2)/A0] × 100(1)

where A0 is the absorbance of a blank group, A1 is the absorbance of the sample, and A2 is the absorbance of the control group.

#### 2.6.2. Superoxide Anion (SOA) Radical Scavenging Assay

The SOA radical scavenging activity was employed according to the previous method [24]. Briefly, a 1-mL sample solution (25, 50, 100, 200, and 400 μg/mL) was mixed with 5-mL Tris-HCl buffer (0.1 M, pH = 7.4) and stood at room temperature for 20 min. Subsequently, the solution was mixed with 1 mL of 25-mM C_6_H_6_O_3_ solution and reacted at room temperature for 4 min. Finally, 8% HCl (0.1 mL) was added to terminate the reaction, and the absorbance of the mixture was measured at 320 nm. The blank group was treated with distilled water instead of the sample solution, and the control group was treated with distilled water instead of C_6_H_6_O_3_ solution.

SOA radical scavenging activity (%) = [A0 − (A1 − A2)]/A0 × 100(2)

where A0 is the absorbance of the sample, A0 is the absorbance of a blank group, and A2 is the absorbance of the control group.

#### 2.6.3. DPPH Radical Scavenging Assay

The DPPH radical scavenging assay was employed according to the description reported by our lab [21]. Briefly, 3 mL of 0.1-mM DPPH solution (DPPH powder was dissolved in 50% ethanol) was mixed with 1 mL of sample solution with different concentrations (25, 50, 100, 200, and 400 μg/mL), respectively, and the mixture was then incubated at room temperature for 25 min in the dark, and the absorbance was measured at 517 nm. The DPPH radical scavenging rate was calculated by the following equation:

DPPH radical scavenging activity (%) = [1 − (A1 − A0)/(A2 − A0)] × 100(3)

where A0 is the absorbance of the background (without DPPH), A1 is the absorbance of the sample group, and A2 is the absorbance of the blank group (without sample).

#### 2.6.4. Hydroxyl Radical (OH¯) Scavenging Assay

The OH¯ scavenging activity was employed according to the previous method [25]. Briefly, 2.2 mL of 9-mM ferrous sulfate, 2.2 mL of 9-mM salicylic acid, and 2.2 mL of 8.8-mM hydrogen peroxide were added to 2.2 mL of the sample solution (25, 50, 100, 200, and 400 μg/mL); then, the mixture was incubated in a 37 °C water bath for 25 min. The absorbance at a wavelength of 510 nm was measured after cooling. The OH¯ radical scavenging rate was calculated by the following equation:

OH¯ radical scavenging rate (%) = [1 − (A2 − A1)/A0] × 100(4)

where A0 is the absorbance of the blank group (without sample), A1 is the absorbance of the background (without hydrogen peroxide), and A2 is the absorbance of the sample group.

### 2.7. In Vitro Hypoglycemic Activities

#### 2.7.1. α-Glucosidase and α-Amylase Inhibiting Assays

The α-glucosidase and α-amylase inhibitory activities of the DTPSs were performed according to the method previously reported by our lab [19] with slight modifications.

##### α-Glucosidase Inhibiting Assay

Briefly, 20-μL α-glucosidase solution (1 U/mL) and 500-μL reduced glutathione (1 mg/L) were added to 2-mL sample solutions (dissolved in 67-mmol/L phosphate buffer solution with pH = 6.8) with different concentrations (25, 50, 100, 200, and 400 μg/mL) and then incubated at 37.5 °C for 10 min. After that, a 200-μL pNPG saturated solution was added to the mixed system and incubated at 37.5 °C for 20 min. Finally, 5 mL of sodium carbonate (1 mol/L) was added to stop the reaction, and the absorbance at a wavelength of 400 nm was measured. The α-glucosidase inhibition rate was calculated by the following equation:

α-glucosidase inhibition rate (%) = [A0 − (A1 − A2)]/A0 × 100(5)

where A0 is the absorbance of the blank group (without sample), A1 is the absorbance of the sample group, and A2 is the absorbance of the background (without enzyme).

##### α-Amylase Inhibiting Assay

In short, a 120-μL pretreated α-amylase solution (working concentration 10 U/mL, dissolved in 0.2-M phosphate buffer, pH = 6.9) was mixed with a 100-μL sample solution (25, 50, 100, 200, and 400 μg/mL) and 80-μL 1% starch solution and then incubated at 37 °C for 5 min. Next, 40 μL of 3,5-dinitrosalicylic acid (DNS) was added and incubated in a boiling water bath for 5 min. After cooling, the volume was fixed to 4 mL, and the absorbance was measured at a wavelength of 540 nm. In the blank control, 0.2 M of phosphate buffer was used instead of the sample solution, and in the background control, 0.2 M of phosphate buffer was used instead of the α-amylase solution. The α-amylase inhibition rate was calculated by the following equation:

α-amylase inhibition rate (%) = [1 − (A2 − A3)/A1] × 100(6)

where A1 is the absorbance of the blank control, A2 is the absorbance of the sample, and A3 is the absorbance of the background.

#### 2.7.2. Glucose Adsorption Capacity (GAC) and Glucose Diffusion and Dialysis Retardation Index (GDRI) Assays

The GAC and GDRI assays were performed regarding the method of Huang et al. [26].

##### GAC Assay

Briefly, a 50-mg polysaccharide sample was mixed with a 5-mL glucose solution (100 mmol/L) and incubated in a water bath at 37 °C for 6 h. After centrifugation (5000× *g*, 10 min), the glucose oxidase method kit was used to determine the glucose concentration in the supernatant. The GAC of the sample was calculated by the following equation:

GAC (mmol/g) = (C0 − C1)/M0(7)

where C0 and C1 are the glucose concentrations (mmol/L) in the solution before and after adsorption, and M0 is the weight of the polysaccharide sample.

##### GDRI Assay

Briefly, a 20-mg polysaccharide sample was added to a 10-mL glucose solution (100 mmol/L) and dialyzed against 200 mL of distilled water in a 37 °C water bath (cutoff Mw 12,000). At different times during the incubation process (15, 30, 60, 90, 120, 150, and 180 min), 1 mL of dialysate was collected, and the glucose concentration was measured by using the glucose oxidase method kit. GDRI was calculated by the following equation:

GDRI (%) = 100 − [(C1/C2) × 100](8)

where C1 is the glucose concentration of the dialysate of the sample mixed with a glucose solution, and C2 is the glucose concentration of the control group.

#### 2.7.3. MTT and Glucose Consumption (GC) Assays

The HepG2 cells were cultured in DMEM medium supplemented with high-glucose (4.5 g/L), FBS (10%), and penicillin–streptomycin (100 U/mL), with a humidified atmosphere (37 °C, 5% CO_2,_ and 95% humidity). The MTT assay of different concentrations of CDTPS (0.25, 0.5, 0.75, 1, 1.25, 1.5, 1.75, and 2 mg/mL) was carried out according to the method described by Qin et al. [27]. The insulin-resistant cell model was established using the method reported by Zheng et al. [28]. Briefly, the cells (10^4^/mL) were colonized in 96-well plates; then, the model group (Control) and sample groups were cultured in DMEM medium with 10^−7^ M recombinant human insulin (Applygen Technologies, Beijing, China) for 48 h, and the blank group (Normal) was replaced with the corresponding concentration of PBS. After that, the original medium was removed, the Control and Normal groups were cultured under their original conditions for 36 h, and the DTPS groups were cultured with 0.75 mg/mL of corresponding polysaccharide samples for 36 h. According to the kit instructions, the GOD-POD method was used to measure the glucose content in the culture medium. The GC in the Control and Normal groups were measured to validate the model; it was regarded as a successful insulin resistance model when the glucose consumption of the two groups had an extremely significant difference (*p* < 0.01).

### 2.8. Statistical Analysis

All statistical measurements were expressed as the mean ± standard deviation. The significant differences between the two groups and among multiple groups were analyzed using the *t*-test and Tukey’s test of a one-way ANOVA, respectively. A *p*-value less than 0.05 was regarded significant. All correlation tests were carried out with Pearson’s correlation analysis.

## 3. Results and Discussion

### 3.1. Model Fitting

**Figure 1:**
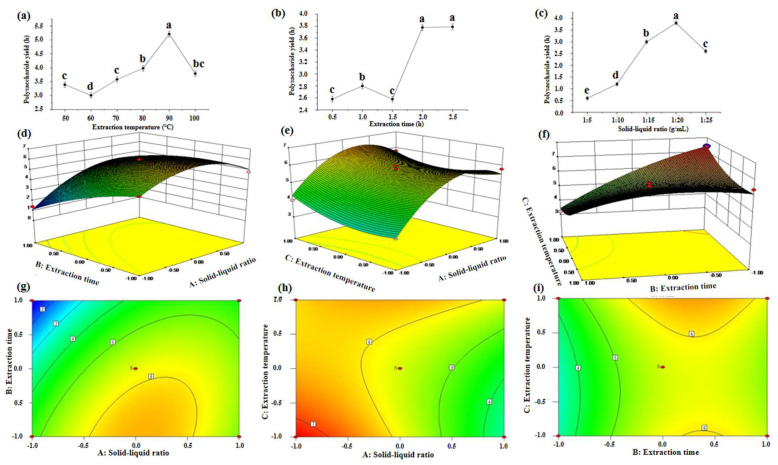
Effects of different single-factor conditions, including the extraction temperature (**a**), extraction time (**b**), and solid-liquid ratio (**c**) on the CDTPS yield. Response surface plots for (**d**,**g**) Y = f (B, A), (**e**,**h**) Y = f (C, A), and (**f**,**i**) Y = f (C, B).

From the single-factor test results (Figure 1a–c), it can be seen that different test factors had a significant impact on the CDTPS yield. According to the results of the single-factor experiment, 17 groups of experiments (Table S3) were designed based on the CCD model. The response range of the CDTPS yield was 1.4~7.4%, suggesting that different factors and levels had a greater impact on the CDTPS yield. The CDTPS yield and experimental factor variables could be explained by the following quadratic regression Equation (9):

Y = 5.66 + 0.9X_1_ − 1.08X_2_ + 0.27X_3_ + 0.75X_1_X_2_ − 0.15X_1_X_3_ + 0.9X_2_X_3_ − 1.38X_1_^2^ − 0.43X_2_^2^ + 0.47X_3_^2^ (9)

where Y is the yield (%) of CDTPS, and X_1_, X_2_, and X_3_ are the coding variables.

The ANOVA analysis from Table S4 shows that the *p*-value of the quadratic regression equation was 0.0001, which showed that the model was sufficiently effective. In addition, the effect of lack-of-fit (*p* > 0.05) was not significant, indicating that the model had a good fit and low experimental error and could analyze and predict the extraction process parameters of CDTPS. Furthermore, the R-squared value was 0.9795, which revealed that the model could explain 97.95% of the variation. The model adjustment coefficient (Adj R-squared) was 0.9531, indicating that the data had high reliability [29]. In the analysis of the influence of various factors, X_1_, X_2_, X_3_, X_1_X_2_, X_2_X_3_, X_12_, X_22_, and X_32_ had remarkable effects on the CDTPS yield, while X_1_X_3_ was not significant (*p* > 0.05). Moreover, from the F value in Table S4, the main factors affecting the CDTPS yield were ranked as X2 > X1 > X3, which was the extraction time > solid–liquid ratio > extraction temperature. Since the three-dimensional response surface can visually display the interactions between two variables, it can be used to determine the optimal level of the two variables (Figure 1d–i). As shown in Figure 1d,g, the interaction between the solid–liquid ratio and extraction time was strong. On the contrary, the interaction between the extraction time and extraction temperature was weak (Figure 1f,i). Besides, it can be seen from Figure 1d,g that the extraction time had the greatest impact on the CDTPS yield (the densest contour and the steepest response surface), which was consistent with the results in the ANOVA analysis. The binomial regression model was used to predict and analyze, and the optimum process conditions for CDTPS extraction were obtained: 2 h for the extraction time, 1:20 for the solid–liquid ratio, and 95 °C for the extraction temperature. Under these conditions, the CDTPS yield was 6.0%. According to the optimal conditions obtained by the analysis and design, the CDTPS yield was obtained (6.067 ± 0.06%) by repeating three times, which was consistent with the predicted value, thus confirming the reliability of the response surface model.

### 3.2. Separation of CDTPS

**Figure 2:**
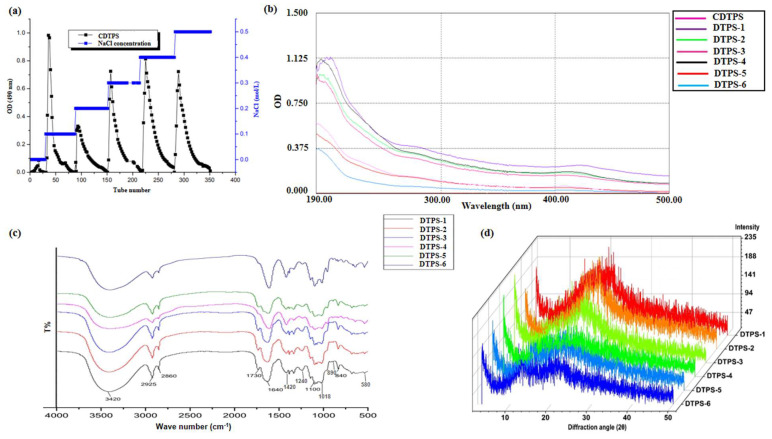
Elution curve (**a**), UV–Vis spectra (**b**), TTIR spectra (**c**), and XRD analysis (**d**) of the DTPSs.

According to the difference in the quantity of electric charge carried by DTPS, CDTPS can be separated by a DEAE Sepharose Fast Flow anionic gel chromatography column. A stepwise elution was performed with PBS (pH = 6.0, 0.02 M) containing different concentrations of NaCl (0, 0.1, 0.2, 0.3, 0.4, and 0.5 M) to obtain six fractions (Figure 2a)—namely, DTPS-1, DTPS-2, DTPS-3, DTPS-4, DTPS-5, and DTPS-6. It can be seen from Table 1 that DTPS-4 had the highest yield, followed by DTPS-6, DTPS-5, and DTPS-1 with the lowest yield. Figure 2b showed no absorption peaks at 260 and 280 nm from the UV spectrum, indicating that there are no free nucleic acids and proteins in CDTPS and the DTPSs [30].

### 3.3. Preliminary Chemical Analysis

Table 1 provided the protein and polyphenol contents in the DTPSs decreased after elution, which indicated that the impurities in polysaccharides could be effectively removed after elution. However, the DTPSs still contained traces of proteins and polyphenols, which was in line with the results reported by Yang et al. that some proteins or polyphenols in DTPSs might exist even after the separation and removal of impurities [18]. Due to the impurity removal steps, we speculate that these trace proteins might be binding proteins bound to polysaccharides. Interestingly, the total sugar content of the DTPSs increased significantly compared with CDTPS, indicating that the elution step could increase the polysaccharides in DTPSs. Furthermore, the contents of protein and polyphenol of the DTPSs and their pH decreased, but the uronic acid increased with the elevation of the concentration of NaCl, which revealed that a high concentration of NaCl might be helpful for the enrichment of uronic acid and the removal of impurities in the elution process. However, the underlying mechanism remains unclear and needs further investigation.

### 3.4. Physicochemical Compositions

**Figure 3:**
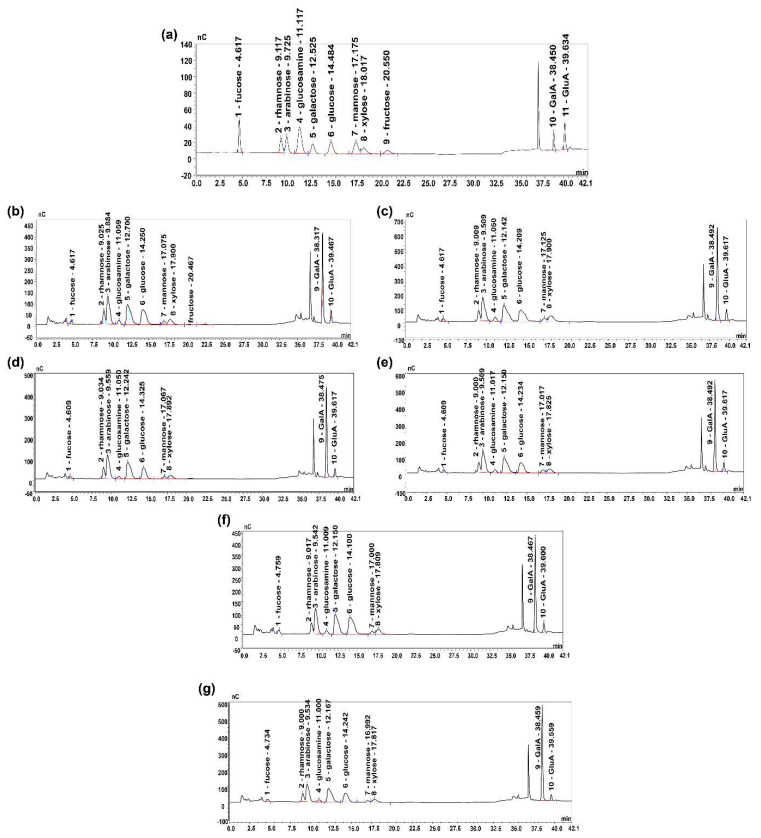
Ion chromatography of the DTPSs. (**a**) Monosaccharide standards, (**b**) DTPS-1, (**c**) DTPS-2, (**d**) DTPS-3, (**e**) DTPS-4, (**f**) DTPS-5, and (**g**) DTPS-6.

The DTPS bioactivity is closely related to the types of its monosaccharide residues; its monosaccharide composition is shown in Table 2. Based on the results of the ion chromatography analysis (Figure 3), six DTPSs contained uniform monosaccharide types (Fucose, Rhamnose, Arabinose, Glucosamine, Galactose, Glucose, Mannose, Xylose, Galacturonic acid, and Glucuronic acid), but the molar concentrations were different. A similar result also was reported by Yang et al. [31]. In addition to the small amount of fructose in DTPS-1, the other fractions did not contain fructose, which indicated that the elution by NaCl might remove fructose from DTPSs. Moreover, the contents of fucose and mannose in each fraction were also low. Surprisingly, the galacturonic acid content in DTPSs was always the highest, further confirming that they were all acidic polysaccharides and might have good bioactivity [32]. Moreover, the glucose content in each fraction remained stable, indicating it might not be affected by the elution concentration of NaCl. The Glucosamine content was the lowest among all the fractions, which further indicated that there might be a few binding proteins in DTPSs. Interestingly, the sum of the Galacturonic acid and Glucuronic acid of DTPS-4 and DTPS-5 in Table 2 was inconsistent with the trend of uronic acid content in Table 1, which indicated that DTPS-4 and DTPS-5 might contain undiscovered mannuronic acid, because it is common in nature. Moreover, the sum of the neutral monosaccharides of DTPSs in Table 2 was also inconsistent with the trend of neutral sugar content in Table 1, which suggested DTPSs might also contain other types of undiscovered monosaccharides. In addition, combined with the previous reports [33,34,35], we speculate that the obtained polysaccharide fraction is a tea polysaccharide conjugate containing pectin, protein, and a small number of other substances, because our preliminary chemical analysis showed that DTPSs were acid polysaccharides containing some other substances. In the monosaccharide composition analysis, the polysaccharide fractions contained a considerable amount of GalA [35,36].

As shown in Table 3 and Figure S5, the Mw distribution of the DTPSs eluted with different NaCl was quite different. All DTPSs had two Mw distributions (Mw > 30 kDa), but the difference in the Mw distribution and ratio was obvious. Since the six polysaccharides obtained were all inhomogeneous, we could not analyze their structures in detail. In addition, since the part with relatively small Mw accounted for a large proportion (>80%) in each DTPS, we mainly analyzed the parts with lower Mw. The Mw distribution of the six DTPSs was as follows: DTPS-2 > DTPS-1 > DTPS-5 > DTPS-3 > DTPS-6 > DTPS-4. Generally, polysaccharides with higher Mw usually have more complex structures and extensive bioactivities. Since polysaccharides are polar macromolecular polymers, there are intramolecular or intermolecular hydrogen bond interactions between a large number of hydroxyl groups on the polysaccharide chain, resulting in various conformations, a high viscosity in the solution, and a more difficult mass transfer, as well as easily affected bioactivity. In contrast, polysaccharides with small Mw can generally exhibit better biological activity, because they can freely pass through biofilms to escape the stress of the immune system [37]. Therefore, DTPSs with lower Mw may have advantages in certain activities.

### 3.5. Structural Characteristics

The results obtained from the FTIR analysis (Figure 2c) show that the DTPSs all contained characteristic absorption peaks of polysaccharides. The strong and broad absorption peaks near 3420 cm^−1^ and 2925 cm^−1^ were caused by O-H and C-H stretching vibrations, respectively [21,38]. The absorption peaks at around 1730 cm^−1^ could be ascribed to the C=O stretching vibration [39]. An intensive absorption peak around 1640 cm^−1^ combined with an absorption peak at 1420 cm^−1^ could be assigned to O-C=O antisymmetric and symmetric stretching vibrations [40] and indicated that they might contain uronic acid, because it is well-known that uronic acid is defined as a lactone compound, and its derivatives formed after the primary hydroxyl group in sugar are oxidized to a carboxyl group. The absorption peaks at 1240 and 840 cm^−1^ could be attributed to the stretching vibration of S=O and the stretching vibration of C–O–S [40,41], and the existence of these two types of vibrations preliminarily suggested that the polysaccharides were sulfated successfully [38]. The absorption peaks at around 1018 cm^−1^ could be ascribed to the stretching vibrations of C-O [40,42].

XRD can be used to characterize the crystalline structure of high-molecular polymers. The crystalline structure of polysaccharides directly determines their tensile strength, flexibility, solubility, and other physical properties. From Figure 2d, it can be seen that DTPS-1, DTPS-2, and DTPS-3 all had obvious single diffraction peaks with rounded and broad intensities near 21°, whereas DTPS-4~6 had two diffraction peaks with a weak intensity, indicating that the crystallinity of DTPS 1~3 might be higher than that of the other fractions. The previous research results in our lab indicated that it was usually difficult for tea polysaccharides to form a large number of single crystals [22]. Therefore, DTPS-1, DTPS-2, and DTPS-3 might coexist in a microcrystalline and amorphous state, while the other fractions were almost in an amorphous state.

**Figure 4:**
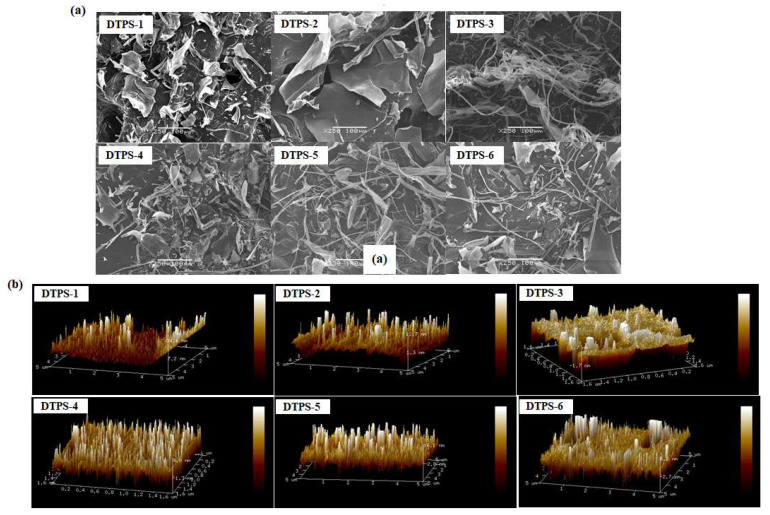
SEM (**a**) and AFM (**b**) images of the DTPSs.

SEM technology can be used to observe the surface, internal morphology, and pore characteristics of polysaccharides [43]. Figure 4a shows that the apparent morphologies of the six DTPSs were different. Specifically, DTPS-1 was mainly irregular flakes with some particles, and the surface was relatively smooth. DTPS-2 was mainly presented with a smooth, dense, and compact flake shape. DTPS-3 was mainly composed of coarse filaments. DTPS-4 mainly exhibited loose, scattered, and irregular rough fragments. DTPS-5 and DTPS-6 mainly presented dense flakes and rough filaments. Generally, a filamentous and granular morphology can increase the specific surface area of the polysaccharide, and the rough and loose structure can enhance the adsorption of the polysaccharide [26].

The AFM images of the DTPSs are shown in Figure 4b. It can be seen that the six DTPSs all showed irregular needle-shaped and rod-shaped morphologies with an arrow-pointed structure, which could be partially explained by the aggregation of the polysaccharide molecules. Studies have shown that acidic polysaccharides rich in hydroxyl and carboxyl groups could cause strong intermolecular and intramolecular interactions with each other, leading to the aggregation of polysaccharides [44]. The ten-point heights of the microcosmic unflatness of DTPS-1~6 were 5.60, 8.46, 3.1, 1.88, 6.28, and 9.65 μm, respectively, and the maximum heights of the contours were 71.3, 124, 47.6, 27.4, 97.5, and 148 μm, respectively. Therein, the surface evenness of DTPS-4 was superior. Studies have shown that the height of a single polysaccharide chain was in the range of 0.1–1 nm [44]. Our results showed that the DTPSs were branched and aggregated (increased viscosity) and might exhibit strong bioactivity due to their certain degree of branching.

**Figure 5:**
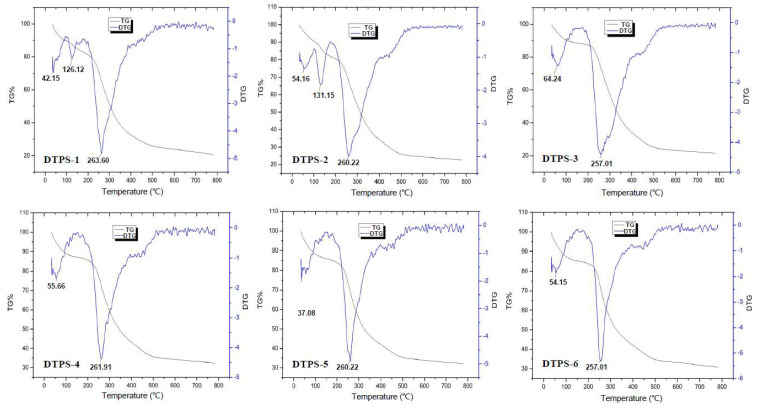
TG and DTG curves of the DTPSs.

Since the thermal stability and thermal decomposition temperature of the substances can be obtained from the TG and DTG curves, we used a thermogravimetric analysis to compare the thermal stability of the DTPSs. It can be seen from the TG curve in Figure 5 that all the DTPSs had weight loss in the temperature ranges of 25–200 °C and 200–600 °C. Less weight loss in the temperature range of 25–200 °C could be attributed to the loss of the physical adsorption of water of the polysaccharide fractions, and more weight loss at 200–600 °C may be due to the depolymerization of the polysaccharides and the cleavage of C-O and C-C in the sugar ring units. The total weight loss rates of DTPS-1~6 were 75.59%, 75.43%, 64.49%, 65.28%, 65.73%, and 66.74%, respectively. The DTG curve showed that DTPS-1 and DTPS-2 had three temperature points with the fastest weight loss rate, while the other fractions had two. These results indicated that the structural stability of DTPS-3~6 might be higher than that of DTPS-1 and DTPS-2. It has been reported that other substances bound to the polysaccharide would affect the thermal stability of the polysaccharide, and molecular conformation, monosaccharide composition, and the structures of the polysaccharide fractions could affect the pyrolysis characteristics. Consequently, these differences in thermal stability might be caused by the distinctions between their chemical compositions and structural characteristics [18,45].

**Figure 6:**
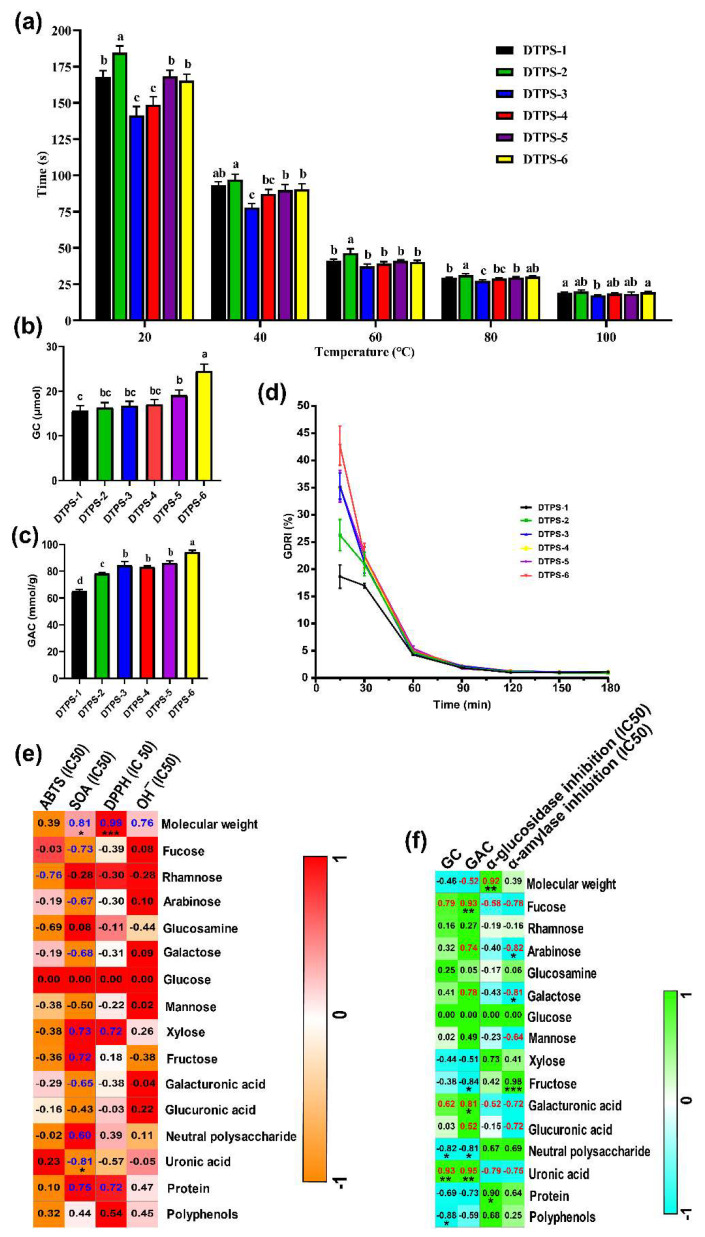
Solubility (**a**), GC (**b**), GAC (**c**), GDRI (**d**), heat map of correlation analysis between the chemical composition and antioxidant activities (**e**), and heat map of the correlation analysis between the chemical composition and hypoglycemic activities (**f**). Different letters represent a significant difference among multiple groups. * *p* < 0.05, ** *p* < 0.01, and *** *p* < 0.001.

To better develop DTPSs into industrialized functional foods, we measured the solubility of the DTPSs at different temperatures. As shown in Figure 6a, the time for the DTPSs to completely dissolve showed a falling trend as the temperature increased. When the temperature outweighed 80 °C, there was almost no difference. At the same temperature, the dissolution time of DTPS-3 was always the shortest because of its coarse filament appearance (Figure 4a). This fragmented and rough appearance greatly increased the surface area of the polysaccharides, which facilitated their dissolution. It can be seen that the order of their solubility was DTPS-3 > DTPS-4 > DTPS-6 > DTPS-5 > DTPS-1 > DTPS-2. Thus, DTPS-3, DTPS-4, and DTPS-6 had potential utilization.

### 3.6. In Vitro Antioxidant Activities

**Figure 7:**
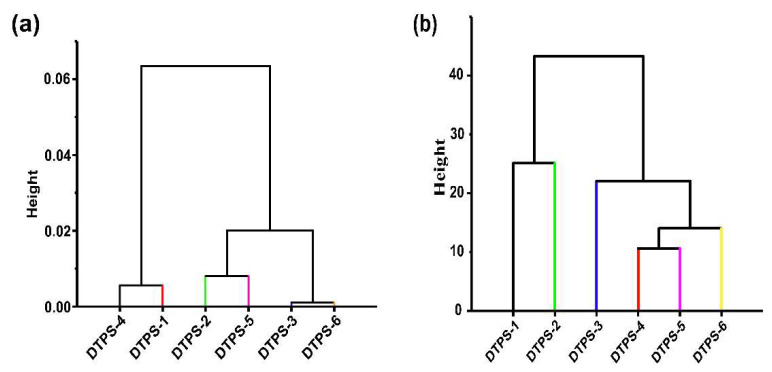
Cluster dendrogram of antioxidant (**a**) and hypoglycemic (**b**) capabilities.

Oxidative stress caused by excessive free radicals in the body has been identified as a key factor in the occurrence and development of diabetes and may cause damage to a variety of tissues and organs [46]. It can be seen from the data in Table 4 that DTPS-4 was the top fraction with the optimum radical scavenging capabilities, including ABTS, SOA, DPPH, and OH¯, whereas a further cluster dendrogram (Figure 7a) of antioxidant capabilities also illustrated this point. Pearson’s correlation analysis (Figure 6e) showed that the ABTS radical scavenging capability was strongly and positively correlated with the rhamnose and glucosamine contents (−0.8 < r < −0.6), and the SOA radical scavenging capability had a significant and positive correlation with the uronic acid content; a significant and negative correlation with Mw; and a strong and positive correlation with fucose, galactose, and galacturonic acid contents (−0.8 < r < −0.6). Moreover, the DPPH radical scavenging capability was significantly and negatively correlated with Mw (r = 0.99), and the DPPH radical scavenging capability was strongly and negatively correlated with Mw (0.6 < r < 0.8). Interestingly, we first found the antioxidant capacity of the DTPSs was significantly related to its Mw and uronic acid contents. A strong relationship between the antioxidant activity and Mw of the polysaccharides has been reported in the literature. Kang et al. found that the antioxidant activity of *maca* polysaccharides with a low Mw was significantly stronger than that of a high Mw [47]. Shi et al. reported that the *Ulva pertusa* polysaccharide with a low Mw was more effective than the high Mw in protecting mice against free radical damage to bone marrow cells and the immune system [48]. These factors may explain the relatively good correlation between the antioxidant activity and Mw of the DTPSs. On the one hand, compared with high-Mw polysaccharides, the viscosity of low-Mw polysaccharides is reduced, making it easier to contact free radicals and achieve the purpose of scavenging free radicals. On the other hand, low-Mw polysaccharides have more reducing ends, which can better react with free radicals to exhibit a quenching effect. In addition to the Mw, prior studies have also indicated a strong correlation between the antioxidant activity and uronic acid content in polysaccharides [49,50]. A possible explanation for this might be that polysaccharides with a higher uronic acid content are more likely to activate anomeric hydrogen atoms to react with free radicals [51]. Contrary to expectations, this study did not find a strong or significant correlation between the radical scavenging capabilities and polyphenol content. Our lab found that the radical scavenging activity of TPS might be attributed to the carboxyl groups in hexuronic acid rather than polyphenols [52].

### 3.7. In Vitro Hypoglycemic Activities of DTPSs

Diabetes is a common endocrine system disease characterized by chronic hyperglycemia in clinical and can cause a series of serious complications [53]. The occurrence of postprandial hyperglycemia is closely related to many factors, like glucose absorption. α-glucosidase and α-amylase are the main enzymes in the small intestine that hydrolyze oligosaccharides and starch; the inhibition of these two enzyme activities contributes to deferring the hydrolysis of starch and oligosaccharides, which can effectively slow down the absorption of glucose in the small intestinal mucosa [54]. Moreover, GAC and GDRI assays are also commonly used to assess the capability of the gastrointestinal tract to postpone glucose absorption [26]. The results of Table 4 indicate that DTPS-4, DTPS-5, and DTPS-6 possessed better α-glucosidase and α-amylase inhibitory activities compared with DTPS-1, DTPS-2, and DTPS-3. From the data of the GAC and GDRI assays (Figure 6c,d), we can see that the top fraction possessing the GAC and GDRI was DTPS-6. To better explore the effect of the DTPSs on glucose absorption and metabolism in the body, we established an insulin-resistant HepG2 cell model to analyze the effects of different DTPSs on the glucose consumption of HepG2 cells. The MTT experiment (Figure S6) showed that CDTPS could not affect the proliferation of HepG2 cells under insulin resistance. The GC result indicated that DTPS-6 showed the optimum GC capability (Figure 6b). A further cluster analysis (Figure 7b) exhibited that the hypoglycemic activity of DTPS-6 could be classified into one type, followed by DTPS-5 and DTPS-4, DTPS-3, DTPS-2, and DTPS-1. A comparison of these results revealed that the top fraction with the hypoglycemic capacity was DTPS-6, followed by DTPS-5, DTPS-4, and DTPS-3. It seems possible that these results were due to the influence of the physicochemical composition and structural characteristics of the DTPSs. In our correlation analysis (Figure 6f), there was a significant negative correlation between α-glucosidase inhibition and Mw and a strong correlation between α-glucosidase inhibition and the uronic acid content. In addition, a significant positive correlation between α-amylase inhibition and the arabinose or galactose content and a strong positive correlation between α-amylase inhibition and some monosaccharide contents, including fucose, mannose, and uronic acid contents, were found. It was found that a high content of monosaccharide composition of arabinose played a role in the glucosidase inhibitory activity of polysaccharides [55]. Similarly, a significant positive correlation was found between GAC and the fucose or uronic acid content, a strong positive correlation was observed between GAC and arabinose or galactose, and a significant positive correlation was presented between GC and the uronic acid content. Previous studies have shown that TPS with relatively low molecular weights exhibited better bioactivities [56], and the findings of the current study were consistent with those of Xu, who found TPS with a small Mw showed better bioactivity, including lowering the blood glucose and antioxidation [57]. Additionally, the high content of uronic acid in polysaccharides has also been pointed out to have superior bioactivity [58,59]. Our previous studies also showed that the inhibitory activity on α-glucosidase by green tea TPS was boosted with the increase of the uronic acid content, and some reports also indicated that the glucosidase inhibition was positively correlated with the uronic acid content in the polysaccharides [60,61]. According to the report by Wang et al., compounds with specific structures of monosaccharide analogs, such as multi-carbon ring compounds with five- or six-membered carbon rings, have significant glucosidase inhibitory activity [19], whereas uronic acid usually presents in the form of pyranose, furanose, and their corresponding lactones with a five- or six-membered carbon ring. Like proteins, some of the active sites in polysaccharides may be combined with some targets in the regulation of the body metabolism, thereby exerting biological functions, and changes in the physicochemical properties of a certain active site may change its bioactivity or biological function [62]; thus, we speculated that the structure of uronic acid may be an important site for DTPS to lower blood glucose. In addition to the physicochemical composition, the structural characteristics also affect hypoglycemic activity. Interestingly, some monosaccharides in DTPS such as fucose, arabinose, galactose, and mannose were found to be associated with hypoglycemic activity for the first time. However, more research on this topic needs to be undertaken before the association between these monosaccharides in DTPS and hypoglycemic activity is more clearly understood. In addition to the physicochemical composition, the structural characteristics also affect hypoglycemic activity. Our results indicated that DTPS-3, DTPS-4, DTPS-5, and DTPS-6 all exhibited superior structural stability and solubility, which contributed to their stable activity. Additionally, their apparent morphology with loose, scattered, and irregular rough fragments or filaments increased their specific surface area, absorbing more glucose, and inhibited the glycosidase activity [26,55].

## 4. Conclusions

The extraction process of CDTPS was optimized, and the extraction yield, chemical composition, and structural and biological properties of the six DTPSs obtained from CDTPS were compared. The results showed that all the DTPSs were acidic polysaccharides, but there were significant differences in their yield, chemical composition, molecular weight, crystalline structure, apparent morphology, and thermal stability. In addition, the correlation analysis of the heat map showed that the uronic acid content and molecular weight might be important factors affecting the antioxidation and hypoglycemic capabilities of the DTPSs. In all, our research interpreted that appropriate methods could be used to extract highly active (antioxidant and hypoglycemic) DTPS from Fuzhuan dark tea to prevent or manage diabetes, thus contributing to a better understanding of the structure–activity relationship of dark tea polysaccharides.

## Figures and Tables

**Table 1:** Chemical composition of CDTPS and its eluted fractions.

Sample	Total Sugar (%)	Uronic Acid (%)	Protein (%)	Polyphenols (%)	pH	Yield (%)
CDTPS	71.20 ± 0.19 ^f^	41.41 ± 0.10 ^c^	0.13 ± 0.02 ^a^	2.69 ± 0.02 ^a^	4.64 ± 0.01 ^bc^	6.07
DTPS-1	80.17 ± 0.14 ^e^	33.06 ± 0.12 ^g^	0.12 ± 0.02 ^a^	1.64 ± 0.01 ^c^	4.77 ± 0.01 ^a^	1.07
DTPS-2	80.53 ± 0.21 ^e^	36.88 ± 0.17 ^f^	0.11 ± 0.03 ^a^	1.94 ± 0.04 ^b^	4.65 ± 0.01 ^b^	3.25
DTPS-3	85.26 ± 0.17 ^c^	39.70 ± 0.12 ^e^	0.11 ± 0.02 ^a^	1.83 ± 0.05 ^bc^	4.62 ± 0.01 ^cd^	2.44
DTPS-4	82.33 ± 0.29 ^d^	40.36 ± 0.22 ^d^	0.05 ± 0.01 ^b^	1.33 ± 0.03 ^d^	4.60 ± 0.01 ^de^	6.27
DTPS-5	88.03 ± 0.28 ^b^	44.12 ± 0.21 ^b^	0.08 ± 0.02 ^ab^	1.73 ± 0.04 ^c^	4.59 ± 0.01 ^ef^	5.03
DTPS-6	88.89 ± 0.17 ^a^	49.53 ± 0.13 ^a^	0.05 ± 0.01 ^b^	0.32 ± 0.02 ^e^	4.57 ± 0.01^f^	5.12

Different letters represent a significant difference among multiple groups.

**Table 2:** Monosaccharide composition (molar %) of different DTPSs.

Samples	DTPS-1	DTPS-2	DTPS-3	DTPS-4	DTPS-5	DTPS-6
Fucose	0.06	0.11	0.14	0.12	0.11	0.18
Rhamnose	0.54	0.50	0.78	0.64	0.36	0.69
Arabinose	0.29	1.16	1.65	1.48	0.84	1.44
Glucosamine	0.08	0.08	0.07	0.09	0.06	0.09
Galactose	0.74	1.90	2.57	2.29	1.47	2.47
Glucose	1.00	1.00	1.00	1.00	1.00	1.00
Mannose	0.08	0.15	0.20	0.19	0.10	0.15
Xylose	0.82	0.95	0.73	0.75	0.51	0.70
Fructose	0.10	ND	ND	ND	ND	ND
Galacturonic acid	1.13	2.92	4.06	3.84	2.05	5.07
Glucuronic acid	0.09	0.25	0.26	0.27	0.15	0.21

ND: Not detected.

**Table 3:** The molecular weight distribution of the DTPSs.

Sample	Mw	Proportion (%)
DTPS-1	24,860,000	8
	989,300	92
DTPS-2	31,400,000	10.5
	1,622,000	89.5
DTPS-3	28,200,000	17.9
	685,200	82.1
DTPS-4	15,670,000	8.5
	318,900	91.5
DTPS-5	26,450,000	12.4
	688,300	87.6
DTPS-6	17,490,000	10.3
	464,400	89.7

Mw: average weight molecular weight.

**Table 4:** Glycosidase inhibition and free radical scavenging activities (IC50) of the DTPSs.

Sample	α-Glucosidase	α-Amylase	ABTS	SOA	DPPH	OH¯
	IC50 (μg/mL)					
DTPS-1	91.42 ± 0.54 ^b^	21.45 ± 0.08 ^a^	570.01 ± 12.18 ^e^	438.54 ± 9.75 ^a^	112.13 ± 4.33 ^b^	52.19 ± 2.92 ^cd^
DTPS-2	102.23 ± 0.87 ^a^	4.45 ± 0.56 ^b^	1063.38 ± 15.67 ^b^	421.90 ± 6.78 ^a^	163.90 ± 13.42 ^a^	92.85 ± 3.52 ^a^
DTPS-3	79.69 ± 2.91 ^c^	1.62 ± 0.24 ^c^	665.61 ± 11.34 ^d^	351.46 ± 6.44 ^a^	93.89 ± 2.07 ^c^	71.59 ± 5.30 ^b^
DTPS-4	57.50 ± 2.76 ^e^	0.06 ± 0.01 ^d^	506.04 ± 13.28 ^f^	342.09 ± 3.57 ^a^	64.37 ± 0.08 ^e^	44.32 ± 0.95 ^d^
DTPS-5	64.12 ± 0.80 ^d^	0.04 ± 0.01 ^d^	1354.71 ± 12.69 ^a^	341.79 ± 4.53 ^a^	84.50 ± 2.02 ^cd^	71.61 ± 3.67 ^b^
DTPS-6	56.08 ± 0.31 ^e^	0.02 ± 0.01 ^d^	721.36 ± 13.21 ^c^	341.34 ± 3.55 ^a^	76.60 ± 3.25 ^de^	60.37 ± 4.58 ^c^

Different letters represent a significant difference among multiple groups.

## Data Availability

The datasets used and/or analyzed during the current study are available from the corresponding author on request.

## Supplementary Materials

The following are available online at https://www.mdpi.com/article/10.3390/foods10102276/s1: Table S1: CCD design factors and levels of response surface in the extraction process, Figure S2: Standard curves of glucose (A), bovine serum albumin (B), galacturonic acid (C) and folin (D), Table S3: Three-factor, three-level CCD design for crude polysaccharide extraction method optimization and the investigated response for experimental results, Table S4: ANOVA for response surface quadratic model, Figure S5: SEC profiles of DTPSs on high performance liquid gel permeation chromatography, Figure S6: Cell proliferation of different concentrations of CDTPS.

## References

[1] Tang G.-Y., Meng X., Gan R.-Y., Zhao C.-N., Liu Q., Feng Y.-B., Li S., Wei X.-L., Atanasov A.G., Corke H. (2019). Health Functions and Related Molecular Mechanisms of Tea Components: An Update Review. Int. J. Mol. Sci..

[2] Lin F.-J., Wei X.-L., Liu H.-Y., Li H., Xia Y., Wu D.-T., Zhang P.-Z., Gandhi G.R., Li H.-B., Gan R.-Y. (2021). State-of-the-art review of dark tea: From chemistry to health benefits. Trends Food Sci. Technol..

[3] Chen G., Xie M., Wan P., Chen D., Dai Z., Ye H., Hu B., Zeng X., Liu Z. (2018). Fuzhuan Brick Tea Polysaccharides Attenuate Metabolic Syndrome in High-Fat Diet Induced Mice in Association with Modulation in the Gut Microbiota. J. Agric. Food Chem..

[4] Xiang X., Xiang Y., Jin S., Wang Z., Xu Y., Su C., Shi Q., Chen C., Yu Q., Song C. (2020). The hypoglycemic effect of extract/fractions from Fuzhuan Brick-Tea in streptozotocin-induced diabetic mice and their active components characterized by LC-QTOF-MS/MS. J. Food Sci..

[5] Lee D.M., Battson M.L., Jarrell D.K., Cox-York K., Foster M.T., Weir T.L., Gentile C.L. (2017). Fuzhuan tea reverses arterial stiffening after modest weight gain in mice. Nutrition.

[6] Wang H., Shi S., Bao B., Li X., Wang S. (2015). Structure characterization of an arabinogalactan from green tea and its anti-diabetic effect. Carbohydr. Polym..

[7] Wei X., Liu Y., Xiao J., Wang Y. (2009). Protective Effects of Tea Polysaccharides and Polyphenols on Skin. J. Agric. Food Chem..

[8] Yang K., Gao Z.-Y., Li T.-Q., Song W., Xiao W., Zheng J., Chen H., Chen G.-H., Zou H.-Y. (2019). Anti-tumor activity and the mechanism of a green tea (*Camellia sinensis*) polysaccharide on prostate cancer. Int. J. Biol. Macromol..

[9] Yuan C., Li Z., Peng F., Xiao F., Ren D., Xue H., Chen T., Mushtaq G., Kamal M.A. (2015). Combination of selenium-enriched green tea polysaccharides and Huo-ji polysaccharides synergistically enhances antioxidant and immune activity in mice. J. Sci. Food Agric..

[10] Chen G., Yuan Q., Saeeduddin M., Ou S., Zeng X., Ye H. (2016). Recent advances in tea polysaccharides: Extraction, purification, physicochemical characterization and bioactivities. Carbohydr. Polym..

[11] Jin F., Jia L.-Y., Tu Y.-Y. (2015). Structural analysis of an acidic polysaccharide isolated from white tea. Food Sci. Biotechnol..

[12] Yin L., Fu S., Wu R., Wei S., Yi J., Zhang L.-M., Yang L. (2020). Chain conformation of an acidic polysaccharide from green tea and related mechanism of α-amylase inhibitory activity. Int. J. Biol. Macromol..

[13] Du L.-L., Fu Q.-Y., Xiang L.-P., Zheng X.-Q., Lu J.-L., Ye J.-H., Li Q.-S., Polito C.A., Liang Y.-R. (2016). Tea Polysaccharides and Their Bioactivities. Molecules.

[14] Chen D., Sun J., Dong W., Shen Y., Xu Z. (2018). Effects of polysaccharides and polyphenolics fractions of Zijuan tea (Camellia sinensis var. kitamura) on α-glucosidase activity and blood glucose level and glucose tolerance of hyperglycaemic mice. Int. J. Food Sci. Technol..

[15] Deng Y.-T., Lin-Shiau S.-Y., Shyur L.-F., Lin J.-K. (2015). Pu-erh tea polysaccharides decrease blood sugar by inhibition of α-glucosidase activity in vitro and in mice. Food Funct..

[16] De La Iglesia R., Loria-Kohen V., Zulet M.A., Martinez J.A., Reglero G., De Molina A.R. (2016). Dietary Strategies Implicated in the Prevention and Treatment of Metabolic Syndrome. Int. J. Mol. Sci..

[17] Chen G., Wang M., Xie M., Wan P., Chen D., Hu B., Ye H., Zeng X., Liu Z. (2018). Evaluation of chemical property, cytotoxicity and antioxidant activity in vitro and in vivo of polysaccharides from Fuzhuan brick teas. Int. J. Biol. Macromol..

[18] Yang X.H., Huang M.J., Qin C.Q., Lv B.Y., Mao Q.L., Liu Z.H. (2017). Structural characterization and evaluation of the an-tioxidant activities of polysaccharides extracted from Qingzhuan brick tea. Int. J. Biol. Macromol..

[19] Zhu J., Chen Z., Zhou H., Yu C., Han Z., Shao S., Hu X., Wei X., Wang Y. (2020). Effects of extraction methods on physicochemical properties and hypoglycemic activities of polysaccharides from coarse green tea. Glycoconj. J..

[20] Blumenkrantz N., Asboe-Hansen G. (1973). New method for quantitative determination of uronic acids. Anal. Biochem..

[21] Chen Z., Zhao Y., Zhang M., Yang X., Yue P., Tang D., Wei X. (2020). Structural characterization and antioxidant activity of a new polysaccharide from Bletilla striata fibrous roots. Carbohydr. Polym..

[22] Zhu J., Yu C., Han Z., Chen Z., Wei X., Wang Y. (2020). Comparative analysis of existence form for selenium and structural characteristics in artificial selenium-enriched and synthetic selenized green tea polysaccharides. Int. J. Biol. Macromol..

[23] Re R., Pellegrini N., Proteggente A., Pannala A., Yang M., Rice-Evans C. (1999). Antioxidant activity applying an improved ABTS radical cation decolorization assay. Free Radic. Biol. Med..

[24] Lin X.C., Huang X.D. (2012). Determination the removing rate of superoxide anion radical by traditional Chinese medicine. Guangzhou Chem..

[25] Gao T., Tang H.L., Luo Z.Y., Luo H.Y., Li M. (2021). Primary structure analysis and antioxidant activity in vitro of crude poly-saccharide from Chuanminshen violaceum. J. Chin. Inst. Food Sci. Technol..

[26] Huang H., Chen J., Chen Y., Xie J., Liu S., Sun N., Hu X., Yu Q. (2021). Modification of tea residue dietary fiber by high-temperature cooking assisted enzymatic method: Structural, physicochemical and functional properties. LWT.

[27] Qin T., Chen J., Wang D., Hu Y., Zhang J., Wang M., Qiu S., Gao Z., Liu R., Yu Y. (2013). Selenylation modification can enhance immune-enhancing activity of Chinese angelica polysaccharide. Carbohydr. Polym..

[28] Zheng X.K., Ke Y.Y., Feng A.Z., Yuan P.P., Zhou J., Yu Y., Wang X.L., Feng W.S. (2016). The mechanism by which amen-toflavone improves insulin resistance in HepG2 cells. Molecules.

[29] Lin L., Xie J., Liu S., Shen M., Tang W., Xie M. (2017). Polysaccharide from Mesona chinensis: Extraction optimization, physicochemical characterizations and antioxidant activities. Int. J. Biol. Macromol..

[30] Tian Y., Zhao Y., Zeng H., Zhang Y., Zheng B. (2016). Structural characterization of a novel neutral polysaccharide from Lentinus giganteus and its antitumor activity through inducing apoptosis. Carbohydr. Polym..

[31] Yang C., He N., Ling X., Ye M., Zhang C., Shao W., Yao C., Wang Z., Li Q. (2008). The isolation and characterization of poly-saccharides from longan pulp. Sep. Purif. Technol..

[32] Zhang Z.J., Wang F.H., Wang M.C., Ma L.P., Ye H., Zeng X.X. (2015). A comparative study of the neutral and acidic poly-saccharides from Allium macrostemon Bunge. Carbohydr. Polym..

[33] Caffall K.H., Mohnen D. (2009). The structure, function, and biosynthesis of plant cell wall pectic polysaccharides. Carbohydr. Res..

[34] Chen H.X., Zhang M., Qu Z.S., Xie B.J. (2007). Compositional analysis and preliminary toxicological evaluation of a tea poly-saccharide conjugate. J. Agric. Food. Chem..

[35] Colodel C., Vriesmann L.C., Petkowicz C.L.D.O. (2018). Cell wall polysaccharides from Ponkan mandarin (Citrus reticulata Blanco cv. Ponkan) peel. Carbohydr. Polym..

[36] Li J., Wang D., Xing X., Cheng T.-J.R., Liang P.-H., Bulone V., Park J.H., Hsieh Y.S. (2019). Structural analysis and biological activity of cell wall polysaccharides extracted from Panax ginseng marc. Int. J. Biol. Macromol..

[37] Li X., Wang L. (2016). Effect of extraction method on structure and antioxidant activity of *Hohenbuehelia serotina polysaccharides*. Int. J. Biol. Macromol..

[38] Wang Z., Xie J., Shen M., Nie S., Xie M. (2018). Sulfated modification of polysaccharides: Synthesis, characterization and bioactivities. Trends Food Sci. Technol..

[39] Zheng T.T., Gu D.H., Wang X.F., Shen X.J., Yan L., Zhang W.J., Pu Y.H., Ge C.R., Fan J.P. (2020). Purification, character-ization and immunomodulatory activity of polysaccharides from *Leccinum crocipodium* (Letellier.) Watliag. Int. J. Biol. Macromol..

[40] Wiercigroch E., Szafraniec E., Czamara K., Pacia M.Z., Majzner K., Kochan K., Kaczor A., Baranska M., Malek K. (2017). Raman and infrared spectroscopy of carbohydrates: A review. Spectrochim. Acta Part A Mol. Biomol. Spectrosc..

[41] Zhang H., Wang J.-Q., Nie S.-P., Wang Y.-X., Cui S.W., Xie M.-Y. (2015). Sulfated modification, characterization and property of a water-insoluble polysaccharide from Ganoderma atrum. Int. J. Biol. Macromol..

[42] Zhang L., Liu X., Wang Y., Liu G., Zhang Z., Zhao Z., Cheng H. (2017). In vitro antioxidative and immunological activities of polysaccharides from Zizyphus Jujuba cv. Muzao. Int. J. Biol. Macromol..

[43] Zhu J., Chen Z., Chen L., Yu C., Wang H., Wei X., Wang Y. (2019). Comparison and structural characterization of polysaccharides from natural and artificial Se-enriched green tea. Int. J. Biol. Macromol..

[44] Gao Y., Zhou Y., Zhang Q., Zhang K., Peng P., Chen L., Xiao B. (2017). Hydrothermal extraction, structural characterization, and inhibition HeLa cells proliferation of functional polysaccharides from Chinese tea Zhongcha 108. J. Funct. Foods.

[45] Cerqueira M., Souza B.W., Simões J., Teixeira J., Domingues M.R., Coimbra M.A., Vicente A.A. (2011). Structural and thermal characterization of galactomannans from non-conventional sources. Carbohydr. Polym..

[46] Halliwell B. (1994). Free radicals, antioxidants, and human disease: Curiosity, cause, or consequence?. Lancet.

[47] Knag C.C., Hao L.M., Zhhang L.M., Zheng Z.Q., Yongwu Y.W. (2018). Isolation, purification and antioxidant activity of pol-ysaccharides from the leaves of maca (Lepidium Meyenii). Int. J. Biol. Macromol..

[48] Shi J.M., Cheng C.L., Zhao H.T., Jing J., Gong N., Lu W.H. (2013). In vivo anti-radiation activities of the Ulva pertusa poly-saccharides and polysaccharide-iron (III) complex. Int. J. Biol. Macromol..

[49] Huang S.-Q., Ding S., Fan L. (2012). Antioxidant activities of five polysaccharides from Inonotus obliquus. Int. J. Biol. Macromol..

[50] Na Y.S., Kim W.J., Kim S.M., Park J.K., Lee S.M., Synytsya A., Park Y.I. (2010). Purification, characterization and immunostimulating activity of water-soluble polysaccharide isolated from Capsosiphon fulvescens. Int. Immunopharmacol..

[51] Wu H., Min T., Li X., Li L., Lai F., Tang Y., Yang X. (2013). Physicochemical properties and antioxidant activities of acidic polysaccharides from wampee seeds. Int. J. Biol. Macromol..

[52] Wang Y., Yang Z., Wei X. (2012). Antioxidant activities potential of tea polysaccharide fractions obtained by ultra filtration. Int. J. Biol. Macromol..

[53] Camer D., Yu Y., Szabo A., Huang X.-F. (2014). The molecular mechanisms underpinning the therapeutic properties of oleanolic acid, its isomer and derivatives for type 2 diabetes and associated complications. Mol. Nutr. Food Res..

[54] Gill T.A., Singer D.S., Thompson J.W. (2006). Purification and analysis of protamine. Process. Biochem..

[55] Zhao C., Li X., Miao J., Jing S., Li X., Huang L., Gao W. (2017). The effect of different extraction techniques on property and bioactivity of polysaccharides from *Dioscorea hemsleyi*. Int. J. Biol. Macromol..

[56] Chen H., Wang Z., Qu Z., Fu L., Dong P., Zhang X. (2009). Physicochemical characterization and antioxidant activity of a polysaccharide isolated from oolong tea. Eur. Food Res. Technol..

[57] Xu L., Chen Y., Chen Z., Gao X., Wang C., Panichayupakaranant P., Chen H. (2020). Ultrafiltration isolation, physicochemical characterization, and antidiabetic activities analysis of polysaccharides from green tea, oolong tea, and black tea. J. Food Sci..

[58] Yao Y., Xue P., Zhu Y., Gao Y., Ren G. (2015). Antioxidant and immunoregulatory activity of polysaccharides from adzuki beans (*Vigna angularis*). Food Res. Int..

[59] Chen Y., Huang Y., Cui Z., Liu J. (2015). Purification, characterization and biological activity of a novel polysaccharide from Inonotus obliquus. Int. J. Biol. Macromol..

[60] Rodrigues D., Freitas A.C., Sousa S., Amorim M., Vasconcelos M.W., da Costa J.P., Silva A.M., Rocha-Santos T.A., Duarte A.C., Gomes A.M. (2017). Chemical and structural characterization of Pholiota nameko extracts with biological properties. Food Chem..

[61] Wang L., Chen C., Zhang B., Huang Q., Fu X., Li C. (2018). Structural characterization of a novel acidic polysaccharide from Rosa roxburghii Tratt fruit and its α-glucosidase inhibitory activity. Food Funct..

[62] Pomin V.H. (2009). Review: An overview about the structure-function relationship of marine sulfated homopolysaccharides with regular chemical structures. Biopolymers.

