# Supplementary material for: Valorization of Polysaccharides Obtained from Dark Tea: Preparation, Physicochemical, Antioxidant, and Hypoglycemic Properties
Source: Foods. 2021 Sep 26;10(10):2276. doi: 10.3390/foods10102276 (PMC8535028; doi:10.3390/foods10102276)
Supplement: Supplementary file 1 [file foods-10-02276-s001.zip › foods-1368874-supplementary.pdf]

Table S1. CCD design factors and levels of response surface in the extraction process.

Factors	Levels		
	-1	0	1
A. Solid-liquid ratio (g/mL)	1:10	1:15	1:20
B. Extraction time (h)	1.5	2	2.5
C. Extraction temperature (℃)	80	90	100

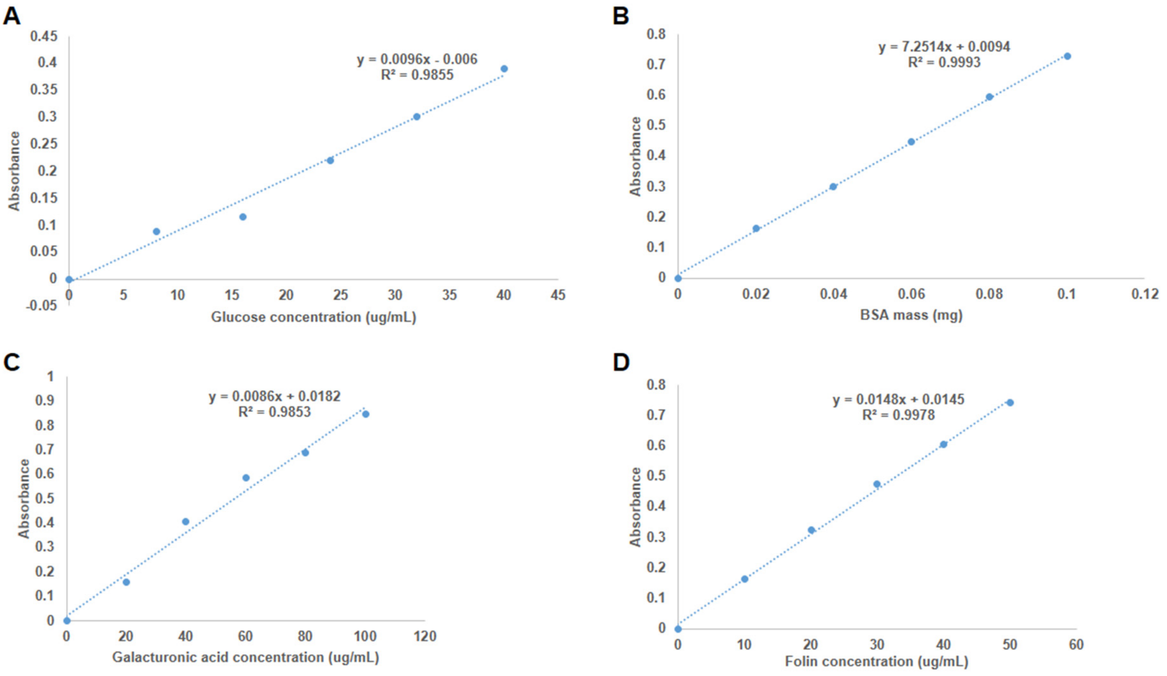

Figure S2. Standard curves of glucose (A), bovine serum albumin (B), galacturonic acid (C) and folin (D).

Table S3. Three-factor, three-level CCD design for crude polysaccharide extraction method optimization and the investigated response for experimental results.

Run No.	A	B	C	Polysaccharide yield (%)
	X_1	X_2	X_3	
1	-1	0	1	4.0
2	0	-1	1	6.4
3	-1	1	0	1.4

4	0	1	1	5.8
5	1	0	1	5.8
6	0	0	0	5.4
7	1	1	0	4.4
8	0	0	0	6.0
9	0	0	0	6.1
10	0	0	0	6.0
11	0	-1	-1	7.4
12	-1	-1	0	4.8
13	-1	0	-1	3.4
14	0	1	-1	3.2
15	1	0	-1	5.8
16	1	-1	0	4.8
17	0	0	0	6.1

Table S4. ANOVA for response surface quadratic model.

Source	Sun of Squares	df	Mean Square	F value	P value	Significance[a]
Model	31.57	9	3.51	37.10	<0.0001	**
X_1	6.48	1	6.48	68.52	<0.0001	**
X_2	9.24	1	9.24	97.76	<0.0001	**
X_3	0.61	1	0.61	6.40	0.0393	*
X_1X_2	2.25	1	2.25	23.79	0.0018	**
X_1X_3	0.09	1	0.09	0.95	0.3618	
X_2X_3	3.24	1	3.24	34.26	0.0006	**
X_1^2	8.02	1	8.02	84.79	<0.0001	**
X_2^2	0.78	1	0.78	8.23	0.0240	*
X_3^2	0.93	1	0.93	9.83	0.0165	*
Residual	0.66	7	0.95			
Lack of Fit	0.43	3	0.14	2.47	0.2013	

Pure Error	0.23	4	0.058
Cor Total	32.24	16	
R-Squared	0.9795		
Adj R-Squared	0.9531		

[a] Significance: *p<0.05, **p<0.01.

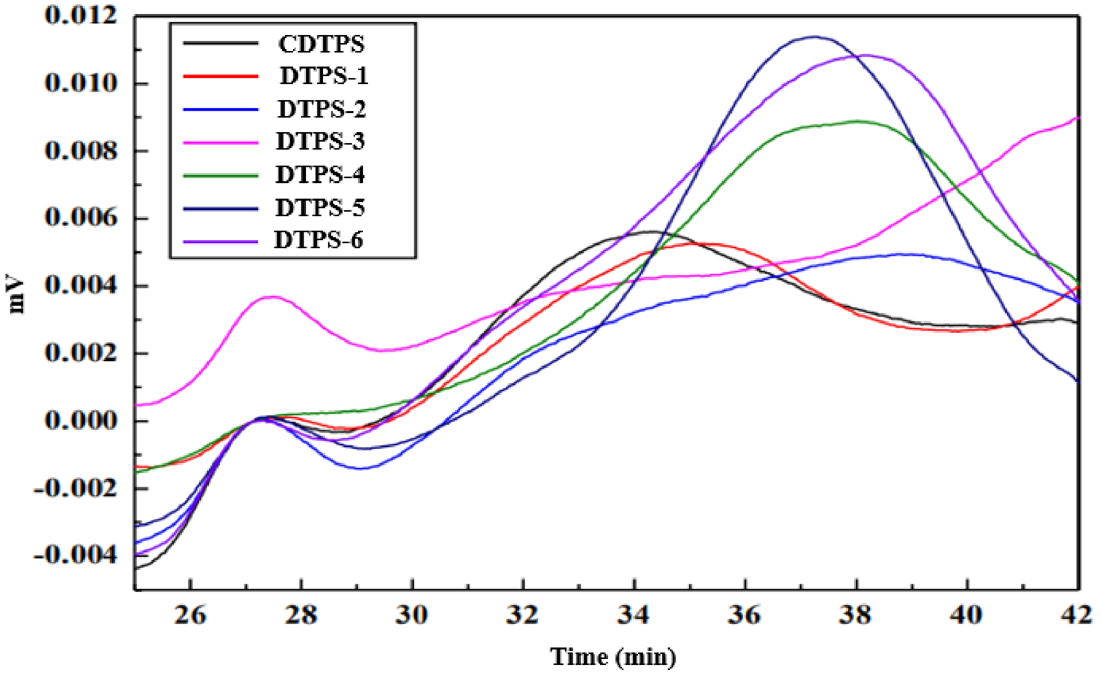

Figure S5. SEC profiles of DTPSs on high performance liquid gel permeation chromatography.

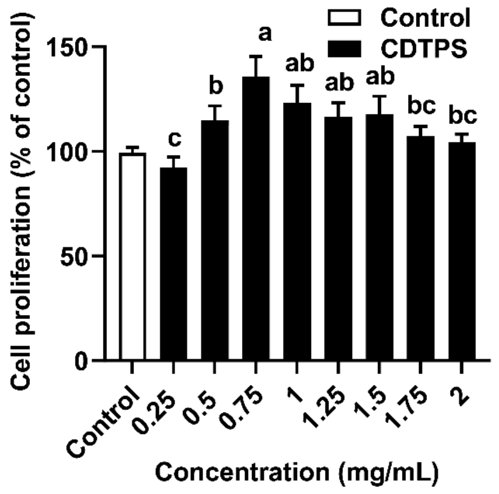

Figure S6. Cell proliferation of different concentrations of CDTPS.
